# Incidence and predictors of recurrent acute coronary syndrome among adult patients with acute coronary syndrome in West Amhara, Ethiopia: a multicenter retrospective follow-up study

**DOI:** 10.3389/fcvm.2023.1234239

**Published:** 2023-10-16

**Authors:** Addis Wondmagegn Alamaw, Tseganesh Asefa, Gebremeskel Kibret Abebe, Alemu Birara Zemariam, Bikis Liyew

**Affiliations:** ^1^ Department of Emergency and Critical Care Nursing, School of Nursing, College of Medicine and Health Sciences, Woldia University, Woldia, Ethiopia; ^2^ Department of Medical Nursing, College of Medicine and Health Sciences, University of Gondar, Gondar, Ethiopia; ^3^ Department of Pediatrics and Child Health, School of Nursing, College of Medicine and Health Science, Woldia University, Woldia, Ethiopia; ^4^ Department of Emergency and Critical Care Nursing, School of Nursing, College of Medicine and Health Sciences, University of Gondar, Gondar, Ethiopia

**Keywords:** parametric model, recurrent acute coronary syndrome, survival analysis, Weibull regression model, incidence rate

## Abstract

**Introduction:**

Acute coronary syndrome (ACS) is the most common cause of morbidity and mortality in patients with coronary heart disease. Furthermore, the recurrence of this problem has significant adverse outcomes. However, there is insufficient information pertaining to this problem in Ethiopia; hence, this study aims to assess the incidence rate and identify the predictors of ACS recurrence in the West Amhara region.

**Methods:**

A retrospective follow-up study was conducted among 469 patients diagnosed with primary ACS. Data from the patient chart were collected using a pre-tested structured data extraction tool. The study employed the Weibull regression analysis model, and the effect size was measured using an adjusted hazard ratio (HR) with a 95% confidence interval (CI). The statistical significance of the findings was established based on a *p*-value <0.05.

**Result:**

A total of 429 patients were included in the final analysis [average age, 60 ± 13.9 years; and 245 (57.1%) men]. A total of 53 patients (12.35%; 95% CI: 9.55%–15.83%) experienced recurrent ACS. The overall risk time was found to be 93,914 days (3,130.47 months), and the recurrence rate was 17/1,000 patients/month. The identified predictors were the typical symptoms of ACS such as syncope (HR: 3.54, *p* = 0.013), fatigue (HR: 5.23, *p* < 0.001), history of chronic kidney disease (HR: 8.22, *p* < 0.001), left ventricular ejection fraction of <40% (HR: 2.34, *p* = 0.009), not taking in-hospital treatments [aspirin (HR: 9.22, *p* < 0.001), clopidogrel (HR: 4.11, *p* = 0.001), statins (HR: 2.74, *p* = 0.012)], and medication at discharge [statins (HR: 4.56, *p* < 0.001)].

**Conclusion:**

This study found a higher incidence rate of recurrent ACS. Hence, the implementation of guideline-recommended anti-ischemic treatment should be strengthened.

## Introduction

Acute coronary syndrome (ACS) is a general term used to describe a spectrum of conditions that are consistent with acute ischemia or infarction, which is characterized by an abrupt decrease in coronary blood flow (1). The resulting undersupply of blood and oxygen to the heart itself can lead to a range of heart conditions, ranging from chest pain to a heart attack/myocardial infarction (MI), during which the heart is damaged (2–4).

According to a report by the British Heart Foundation, Africa had a total of 58 million cases of heart and circulatory diseases, resulting in 1.7 million deaths attributable to these diseases (5). Despite the decline in mortality rates due to ischemic heart disease (IHD) in high-income countries, its trend is increasing in low-income countries from 243,000 in 2000 to 304,000 in 2010, and further increased to 379,000 in 2019. Furthermore, over three-fourth of the global fatalities resulting from cardiovascular diseases (CVDs) occurred in developing countries (4, 6, 7). The economic burden of coronary heart disease (CHD) is also drastically increasing. According to a report conducted in the United States, it is estimated that the indirect costs associated with CVD are projected to increase by 52% (from $202.5 billion to $308.2 billion) from 2013 to 2030. Approximately 43% of the total indirect cost is predicted to be attributed to CHD, making it the condition with the highest indirect expenses (8).

Recurrent ischemia following the initial presentation of an ACS is indicative of an adverse outcome and has major implications for the allocation of healthcare resources. The prevalence of recurrent ACS in developed countries nowadays has become a major concern due to its increasing incidence rate, and it was associated with high adverse outcomes, including stroke, congestive heart failure, CHF), cardiogenic shock, and death (9–15). Recurrent ACS was found to be associated with an increase in mortality rates immediately after the adverse event. Specifically, the hazard ratio for mortality during the first day following a recurrent MI was observed to exceed 14 times. Subsequently, the risk of mortality was reduced but still remained significantly higher (five times) between 1 day and 1 year following a recurrent MI (12). There was a significant difference in death for recurrent ACS compared with death among patients without recurrent ACS (5.2% vs. 2.2% at *p* < 0.01) (16). Pooled data indicate that the patients aged 45 years or older had a mortality rate of 19% for men and 26% for women within the first year. After a period of 5 years following the initial occurrence of MI, the mortality rates for men and women were recorded at 36% and 47%, respectively (17).

Studies from developed nations reveal that the readmission rate due to recurrent ACS has increased from time to time. The study findings show that the readmission rate increased as the time interval between the first ACS discharge and readmission time increased, ranging from 11.7% to 61.7% within the first 30 days and the first year following discharge (10, 13, 15, 18). Furthermore, the median time for readmission was found to be 0.8 months (18).

The prevalence of ACS in Africa has also become increasingly high, as evidenced by available data. In addition, there has been a concurrent rise in the rates of re-infarction and recurrence associated with ACS. According to a study on treatment outcomes of ACS in different sub-Saharan African countries, the re-infarction rate in Kenya was found to be 6.6% (19).

A study conducted in Ethiopia examined the prevalence of CVD among adults at six university referral hospitals. The findings revealed that IHD ranked among the top three types of CVD, accounting for 11.5% (20). In addition, re-infarction ACS was observed in 14.6% of the patients in Mekelle, Ethiopia (21).

In general, the available data show that recurrent ACS has become a challenging problem worldwide. In recent decades, various organizations such as the American College of Cardiology Foundation/American Heart Association (AHA), British Heart Foundation, and other national cardiac associations have actively engaged in addressing this issue by implementing treatment guidelines and secondary prevention measures.

Despite the efforts of national and international organizations to develop prevention and treatment guidelines for ACS, this problem remains a prominent issue worldwide. Despite the existence of established guidelines developed for ACS in developed nations, which have been adopted and implemented in many countries worldwide, ACS has also become a significant concern in developing nations, including in African countries. However, the issue of recurrent ACS in developing nations has not been thoroughly examined in terms of the magnitude of the problem and effective management strategies. Furthermore, the recurrence of this problem has been observed to result in high adverse outcomes, as evidenced by various studies conducted worldwide. However, the extent of this problem in Ethiopia remains mostly unexplored.

This study includes the majority of studies conducted on myocardial infarction, either ST segment elevated myocardial infarction (STEMI) or non-ST segment elevated myocardial infarction (NSTEMI). Furthermore, we incorporated typical symptoms of ACS as individual and independent predictors in this study, which were previously referred to generally as “having typical symptoms” in prior research. Therefore, it is important to study the incidence rate of recurrent ACS and its predictors within the specific context of developing nations. This approach may help in implementing measures at the source of the problem.

Because there is insufficient information regarding this problem in Ethiopia, understanding its incidence and predictors is crucial in order to take secondary preventative measures against its adverse outcomes. Furthermore, it will serve as baseline data for future research.

## Material and methods

### Study design and period

An institutional-based retrospective follow-up study with a record review of patients admitted with the diagnosis of acute coronary syndrome from 1 January 2017 to 31 December 2021 was conducted.

### Study area

The study was conducted in the Comprehensive Specialized Hospitals located in West Amhara, specifically Debre Markos Comprehensive Specialized Hospital, Tibebe Ghion Comprehensive Hospital, Felege Hiwot Comprehensive Specialized Hospital, Debre Tabor Comprehensive Specialized Hospital, and University of Gondar Comprehensive Specialized Hospital (UoGCSH). These hospitals are among the eight comprehensive specialized hospitals found in the region and serve more than 3.5 million people from each town and adjacent catchment areas. The hospitals provide multidimensional aspects of care for clients, including outpatient, inpatient, emergency, intensive care unit, and other services. Most patients with the diagnosis of ACS are admitted to the medical intensive care unit (MICU). However, due to the limited number of beds, a significant number of patients receive treatment either in the emergency department or in the general wards.

### Population

#### Source population

The source population of this study includes all ACS patients receiving care at the comprehensive specialized hospitals in West Amhara. The study population comprises all patients diagnosed with primary ACS who were registered in the admission registration book at the Comprehensive Specialized Hospitals in West Amhara from 1 January 2017 to 31 December 2021.

#### Eligibility criteria

The study included all individuals diagnosed with either STEMI or NTEMI who were admitted to the hospital as primary ACS patients. The inclusion criteria required that these individuals had fully recovered and were discharged alive between 1 January 2017 and 31 December 2021. Patients with incomplete medical records, missing time variables, those who were transferred in, those without at least one follow-up, and those who developed re-infarction were excluded from the study.

#### Sample size determination

Given the absence of any existing research conducted in Ethiopia on this topic, we have undertaken a pilot study. The pilot study was conducted at UoGCSH, serving as an internal pilot study with a sample size of 80 charts of ACS patients. The study revealed that the incidence recurrence rate was 11.25%. Subsequently, the minimum sample size required was calculated using a formula for a single population proportion by considering the following statistical assumptions: *p* = 0.1125, Z*α*/2 = corresponding *Z* score of 95% CI and *d* = margin of error (3%).

$$n=z{\left(\frac{a}{2}\right)}^{2}\frac{\;p(1-p)}{d^{2}}$$

Single population proportion formula *n* = (1.96)^2^ × 0.1125 × 0.8875/(0.03)^2^ = 426. After assuming a 10% margin of error to account for the incomplete chart, the determined minimum sample size required was 469.

#### Sampling procedure and sampling technique

The study was conducted in all five comprehensive specialized hospitals located in the West Amhara district. The method for proportional allocation was utilized for each hospital. The study samples were obtained by identifying the chart numbers of all patients diagnosed with ACS who were admitted to the emergency department, MICU, and medical ward at the selected hospitals. Only patients who were discharged alive after recovering from ACS between 1 January 2017 and 31 December 2021 were included; the chart numbers were retrieved from the registry book. Then, by compiling a list of the index chart numbers of the ACS patients, systematic random sampling methods were used to select a sample of 469 charts. The k interval for each hospital was determined by dividing the total study population of each hospital by the sample population in those hospitals. The selected ACS patient was retrospectively monitored for a maximum of 1 year.

### Operational and standard definition

**Acute coronary syndrome**: For this study, ACS encompasses both myocardial infarction (STEMI and NSTEMI). **STEMI** is characterized by new ST elevation at the J point in two contiguous leads with the cutoff points of ≥0.1 mV in all leads other than the V2–V3 leads. In the V2–V3 leads, the following cutoff points apply: ≥0.2 mV in men aged ≥40 years, ≥0.25 mV in men aged <40 years, or ≥0.15 mV in women (22). **NSTEMI** is characterized by the presence of new horizontal or down-sloping ST depression of ≥0.05 mV in two contiguous leads and/or T-wave inversion of ≥0.1 mV in two contiguous leads with prominent R wave or an R/S ratio of >1 (22). **Chronic kidney disease (CKD)** is defined as a condition of decreased kidney function as indicated by a glomerular filtration rate (GFR) of less than 60 ml/min per 1.73 m^2^, or the presence of markers indicating kidney damage, or both, for a duration of at least 3 months, regardless of the underlying cause (23). **Renal dysfunction** is defined as having blood urea nitrogen levels exceeding 40 mg/dl or creatinine levels exceeding 2.5 mg/dl (9). **Recurrent myocardial infarction (re-MI)** is specifically characterized as MI events occurring beyond a period of 28 days following the initial index MI event. However, MI that occurs within 28 days of the initial MI event is classified as a case of re-infarction (24). In this study, **recurrent ACS** refers to any acute coronary event that occurs after 28 days following the initial incident of ACS (25). **Primary ACS** is used to describe ACS that is diagnosed for the first time during the study period. The **left ventricular ejection fraction (LVEF)** is categorized as either ≥40% and <40% (26). The term **Recovery** is used to refer to patients who were declared recovered during their admission. The term **Censored Cases** refers to cases where ACS patients died after discharge, opted out of follow-up, were transferred out, or did not develop any outcomes. **Event** refers to recurrent acute coronary syndrome that occurs after 28 days of recovery and successful discharge. **Follow-up time** was determined by measuring the time from baseline to the earliest date at which a patient experienced an outcome.

### Data collection tools and procedure

A structured data abstraction tool was adapted from different studies and used to obtain information from the chart (21, 27–29). The data were obtained by a group of six BSc nurses who underwent training and conducted the collection process from 12 May 2022 to 6 June 2022. The investigator facilitated the process and was designated as the supervisor of the data collectors. The accuracy of the data was verified daily.

### Data quality control

To maintain the quality of the data, a pretest was conducted at UoGCSH, which involved a random selection of 15 charts to assess the data abstraction checklist, as well as the quality of the instrument and the completeness of the variables. The pretest results were utilized to inform and implement necessary corrections and modifications.

### Data processing and analysis

Data were coded and entered into Epi info version 7 statistical software and exported to STATA version 14 software for data cleaning, checking, and analysis. The descriptive statistics for various variables were presented by text, frequency, cross-tabulations, pie charts, and bar charts. The parametric survival analysis model (Weibull regression) was implemented to assess the statistical significance of the bivariable analysis. The multivariable analysis included variables with a *p*-value ≤ 0.25 from the bivariable analysis. Kaplan–Meier curves were used to estimate the recurrence-free survival rate and statistical log rank. The proportional hazard assumptions were evaluated using global testing. The multivariable analysis employing Weibull regression was utilized to determine the adjusted hazard ratio (AHR) with a 95% confidence interval (CI) and a significance level of *p* < 0.05 to identify statistically significant predictors of the outcome variable.

## Result

### Socio-demographic characteristics of ACS patients

Out of the 469 patient records of acute coronary syndrome that were reviewed, a total of 429 records were included in the final analysis. Among the participants, more than half (57.1%) of the study participants were male, and the majority (65%) were from urban areas. The mean age of the participants at the time of follow-up initiation was 60 ± 13.9 years. The mean age of patients without recurrent ACS was 58.98 ± 13.47 years, while the mean age of patients who experienced recurrent AMI was 67.25 ± 14.81% years (Table 1).

**Table 1:** Baseline socio-demographic characteristics of acute coronary syndrome patients in West Amhara comprehensive specialized hospitals, Amhara, Ethiopia, 2022.

Variables	Category	Total *N* = 429	Outcomes	
			Censored	Recurrent
Age (years)	Mean, SD	60 ± 13.89	58.98 ± 13.47	67.25 ± 14.81
Age categorized (years)	18–44	58 (13.5%)	53 (91.4)	5 (8.6)
	45–64	204 (47.6%)	191 (93.6)	13 (6.4)
	65–74	86 (20.7)	74 (86.0)	12 (14.0)
	≥75	81 (18.9%)	58 (71.6)	23 (28.4)
Sex	Male	245 (57.1%)	215 (87.8)	30 (12.2)
	Female	184 (42.9%)	161 (87.5)	23 (12.5)
Residence	Urban	279 (65%)	243 (87.1)	36 (12.9)
	Rural	150 (35%)	133 (88.7)	17 (11.3)

### Baseline clinical characteristics and diagnostic tests

#### Baseline vital sign and presentation symptoms

Among the 429 patients diagnosed with ACS, 346 (80.7%) of them experienced chest pain as their initial symptom upon presentation. Subsequently, 46 (13.3%) of those with chest pain developed recurrent ACS. Other frequently observed symptoms were shortness of breath, cough, diaphoresis, and vomiting with a proportion of 54.1%, 42.2%, 27%, and 24.7%, respectively. The baseline vital sign results included a median systolic blood pressure of 120 mmHg (IQR: 110–140), pulse rate 87 beats/min (IQR: 78–100), and random blood sugar of 159.6 mg/dl (95% CI: 151.5–167.6 mg/dl) (Table 2).

**Table 2:** Baseline clinical characteristics of ACS patients in West Amhara comprehensive specialized hospitals, Amhara, Ethiopia, 2022.

Variables	Category	Total	Outcomes	
			Censored	Recurrent
Systolic blood pressure (mmHg)	Median (IQR)	120 (110–140)	120 (110–140)	120 (100–150)
Diastolic blood pressure (mmHg)	Median (IQR)	78 (70–86)	79 (70–85)	70 (70–90)
Pulse rate (beats/min)	Median (IQR)	87 (78–100)	87 (78–100)	84 (79–95)
Chest pain	No	82 (19.1%)	75 (91.5%)	7 (8.5%)
	Yes	347 (80.9%)	301 (86.7%)	46 (13.3%)
Shortness of breathing	No	195 (45.5%)	178 (91.3%)	17 (8.7%)
	Yes	234 (54.5%)	198 (84.6%)	34 (15.4%)
Cough	No	248 (57.8%)	219 (88.3%)	29 (11.7%)
	Yes	181 (42.2%)	157 (86.7%)	24 (13.3%)
Nausea	No	372 (86.7%)	326 (87.6%)	46 (12.4%)
	Yes	57 (13.3%)	50 (87.7%)	7 (12.3%)
Vomiting	No	323 (75.3%)	280 (86.7%)	43 (13.3%)
	Yes	106 (24.7%)	96 (90.6%)	10 (9.4%)
Diaphoresis	No	313 (72.9%)	280 (89.5%)	33 (10.5%)
	Yes	116 (27.1%)	96 (82.1%)	21 (17.9%)
Syncope	No	403 (93.9%)	357 (88.6%)	46 (11.4%)
	Yes	26 (6.1%)	19 (73.1%)	7 (16.9%)
Fatigue	No	390 (90.9%)	346 (88.7%)	44 (11.3%)
	Yes	39 (9.1%)	30 (76.9%)	9 (23.1%)
Others symptoms		6 (1.4%)	5 (83.3%)	1 (16.7%)

#### Baseline history of comorbidities

**Figure 1:**
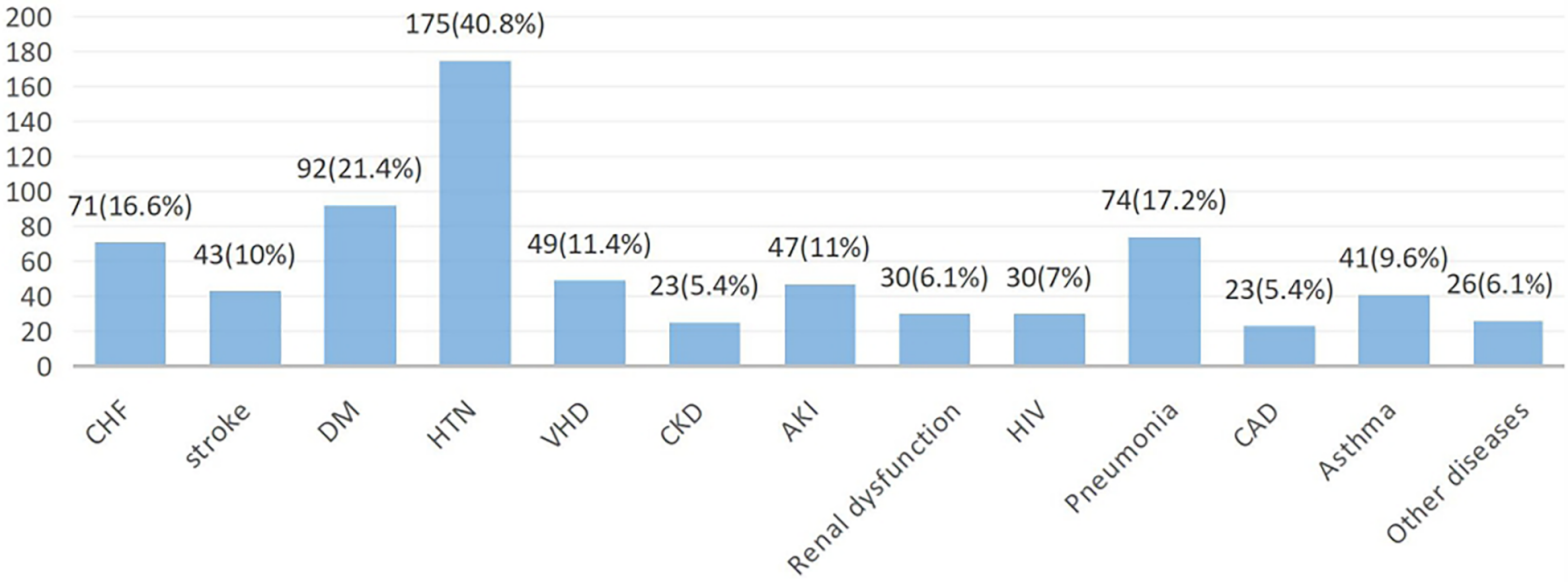
Patterns of comorbidities at primary admission among ACS patients in West Amhara comprehensive specialized hospitals, Amhara, Ethiopia, 2022. CHF, congestive heart failure; DM, diabetes mellitus; HTN, hypertension; VHD, ventricular heart disease; AKI, acute kidney injury; HIV, human immunodeficiency virus; CAD, coronary artery disease.

The most prevalent comorbidities upon admission were hypertension, diabetes mellitus (DM), pneumonia, and CHF accounting for 175 (40.8%), 92 (21.4%), 74 (17.2%), and 71 (16.6%) cases, respectively. Major comorbidities were observed among patients with recurrent ACS, with DM affecting 19.1% and hypertension affecting 15.4% of the patients (Figure 1).

#### Diagnostic tests and diagnosis

Among the 429 ACS cases that were studied, 114 (26.6%) cases exhibited a LVEF of less than 40%. Among these cases, 21.1% experienced recurrent ACS events. A total of 294 (68.5%) patients had left ventricular dysfunction, and 46 (15.6%) patients among this subgroup experienced recurrent ACS. In relation to the lipid profile, it was shown that half (50.3%) of the ACS patients exhibited hyperlipidemia during their initial admission. Among the total follow-up cases, 65.5% were diagnosed with STEMI, whereas the other cases were classified as NSTEMI (Table 3).

**Table 3:** Baseline diagnostic tests and diagnosis of acute coronary syndrome patients in West Amhara comprehensive specialized hospitals, Amhara, Ethiopia, 2022.

Variables	Category	Total, *n* (%)	Outcome	
			Censored, *n* (%)	Recurrent, *n* (%)
Troponin (ng/ml)	Median, IQR	2.9 (0.19–9.32)	2.81 (0.19–9.32)	6.4 (0.172–14.74)
Echocardiography LVEF (%)	<40	114 (26.6)	90 (78.9)	24 (21.1)
	≥40	315 (73.4)	286 (90.8)	29 (9.2)
LV dysfunction	Yes	294 (68.5)	248 (84.4)	46 (15.6)
	No	135 (31.5)	128 (94.80	7 (5.2)
Hyperlipidemia	Yes	216 (50.3)	189 (87.5)	27 (12.5)
	No	213 (49.7)	187 (87.8)	26 (12.2)
Type of MI	STEMI	281 (65.5)	242 (86.1)	39 (13.9)
	NSTEMI	148 (34.5)	134 (90.5)	14 (9.5)

#### In-hospital treatment provided at the primary admission

Regarding the treatment at primary admission, it was observed that aspirin, clopidogrel, statins, beta-blockers, and anticoagulants were not administered for 4.7%, 9.8%, 7.9%, 13.5%, and 17.7% of ACS patients, respectively. Those who did not receive these medications exhibited a higher incidence of recurrent ACS with 55%, 34.5%, 29.4%, 15.5%, and 21.1%, respectively (Table 4).

**Table 4:** In-hospital treatments of acute coronary syndrome patients in West Amhara comprehensive specialized hospitals, Amhara, Ethiopia, 2022.

Treatments	Category	Total, *n* (%)	Outcome	
			Censored, *n* (%)	Recurrent, *n* (%)
Aspirin	Yes	408 (95.4)	367 (90.0)	41 (10.0)
	No	21 (4.7)	9 (42.9)	12 (57.1)
Clopidogrel	Yes	387 (90.2)	351 (90.7)	43 (9.3)
	No	42 (9.8)	25 (76.2)	17 (23.8)
Statins	Yes	393 (92.1)	350 (89.1)	43 (10.9)
	No	36 (7.9)	26 (72.2)	10 (27.8)
ACEIs	Yes	285 (66.4)	248 (87)	37 (13)
	No	144 (35.6)	128 (88.9)	16 (11.1)
Beta-blockers	Yes	371 (86.5)	335 (90.3)	40 (9.7)
	No	58 (13.5)	49 (84.5)	9 (15.5)
Anticoagulant	Yes	353 (82.3)	316 (89.5)	37 (10.5)
	No	76 (17.7)	60 (78.9)	16 (21.1)
Diuretics	Yes	178 (41.5)	154 (86.5)	24 (13.5)
	No	251 (58.5)	222 (88.4)	29 (11.6)

#### Treatment administered upon discharge

Among the 429 ACS patients included in this study, the percentage of patients who have not received evidence-based anti-ischemic medication were aspirin (6.8%), statins (8.6%), clopidogrel (11.9%), beta-blockers (15.2%), and angiotensin converting enzyme inhibitors (ACEIs) (51%). Among those who did not receive those medications (aspirin, statins, and clopidogrel), a higher incidence of recurrent ACS was observed, with 41.4%, 40.5%, and 35.3%, respectively (Table 5).

**Table 5:** Medication prescribed at discharge of ACS patients in West Amhara comprehensive specialized hospitals, Amhara, Ethiopia, 2022.

Variables	Category	Total, *n* (%)	Outcomes	
			Censored, *n* (%)	Recurrent, *n* (%)
Aspirin	Yes	400 (93.2)	359 (89.8)	41 (10.3)
	No	29 (6.8)	17 (58.6)	12 (41.4)
Clopidogrel	Yes	378 (88.1)	343 (90.7)	35 (9.3)
	No	51 (11.9)	33 (64.7)	18 (35.3)
Statins	Yes	392 (92.4)	354 (90.3)	38 (9.7)
	No	37 (8.6%)	22 (59.5)	15 (40.5)
ACEIs	Yes	219 (51.0)	189 (86.3)	30 (13.7)
	No	210 (49.0)	187 (89.0)	23 (11.0)
Beta-blockers	Yes	364 (84.8)	321 (88.2)	43 (11.8)
	No	65 (15.2)	55 (84.6)	10 (15.4)
Diuretics	Yes	111 (25.9)	97 (87.4)	14 (12.6)
	No	318 (74.1)	279 (87.7)	39 (12.3)

### Incidence and survival pattern of ACS patients

**Figure 2:**
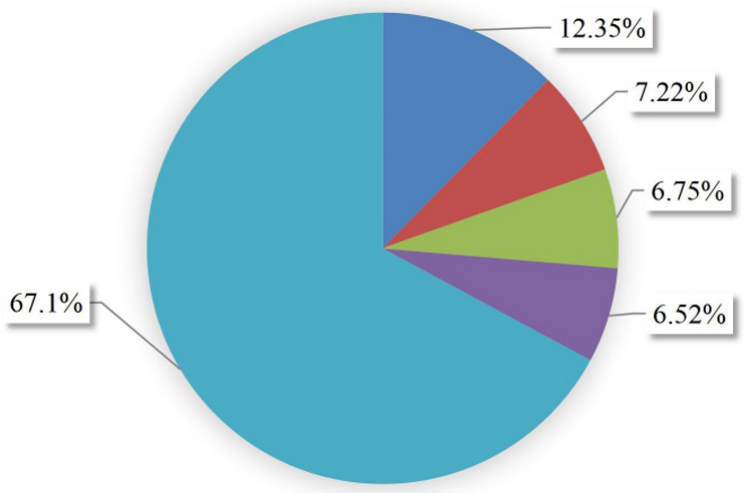
Outcomes proportion of follow-up patients in West Amhara comprehensive specialized hospitals, Amhara, Ethiopia, 2022.

With the a minimum follow-up period of 30 days and a maximum follow-up of 365 days, a total of 53 (12.35%, 95% CI: 9.55%–15.83%) individuals experienced recurrent ACS, while 7.22% of the participants were dead, 6.75% withdrew from follow-up, and 6.52% transferred out. The remaining two-third (67.1%) did not develop any outcome (Figure 2). The total duration at risk for 429 ACS patients was 3,130.467 months (93,914 days). The incidence rate of recurrent ACS was 0.01693 (95% CI: 0.0129–0.0222) per person-month [16.93 Re-ACS/1,000 person-month] or 5.6 (95% CI: 4.3–7.4) per 10,000 person-day observation among follow-up ACS patients. The incidence rates observed at 60, 90,180, and 365 days were 4.02, 9.53, 6.55, and 4.98 per 10,000 person-day observation, respectively. The probability of survival without recurrence at 60, 90, 180, and 365 days following discharge were 0.978, 0.950, 0.893, and 0.817, respectively.

### Failure function

**Figure 3:**
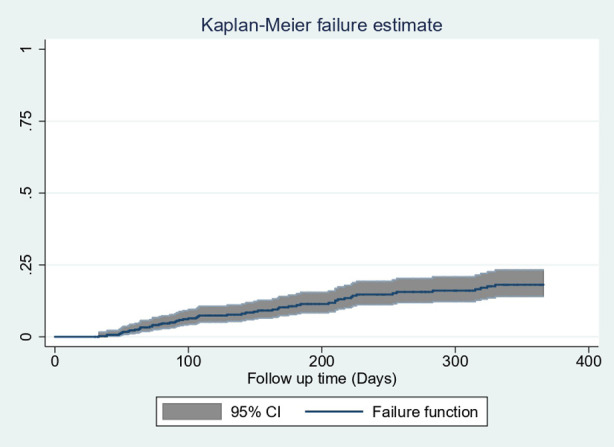
Overall Kaplan–Meier estimation of failure function with 95% confidence intervals of follow-up patients in West Amhara comprehensive specialized hospitals, Amhara, Ethiopia, 2022.

The restricted mean survival time was 328.8 days with 95% CI (319.3–338.3 days). The cumulative probability of failure at 60, 90, 180, and 365 days was 0.022, 0.0499, 0.107, and 0.182, respectively (Figure 3).

### Survival functions of predictors

The survival curves of different predictor variables were tested for equality using the log-rank test. The statistics and the survival curve showed that there were significant differences in the survival functions of different categorical variables. These variables were syncope, fatigue, history of CKD, LVEF, administering aspirin, clopidogrel, and statins during hospitalization, and administering statins at discharge.

**Figure 4:**
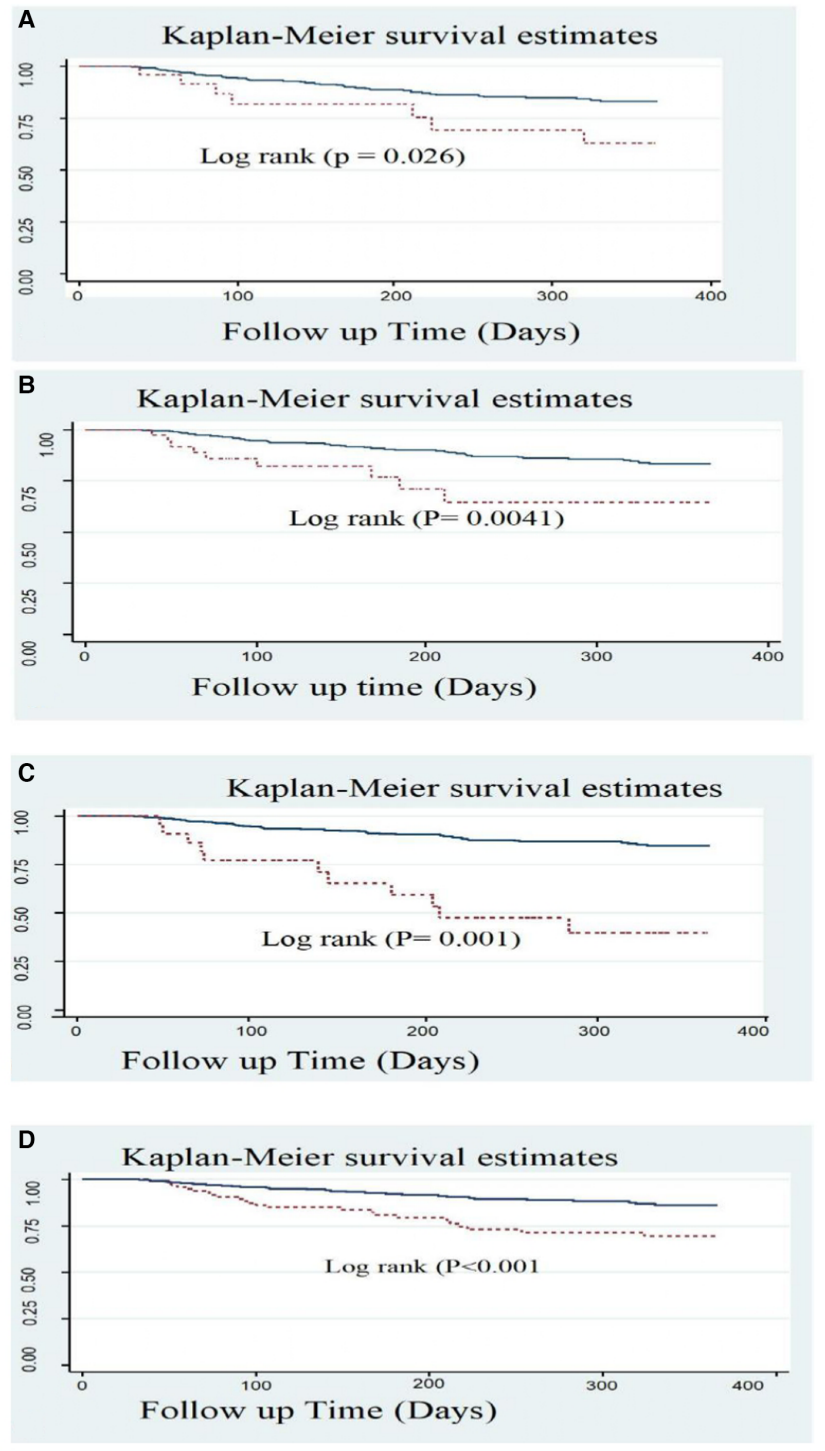
Kaplan Meier survival estimate of ACS patients with typical symptoms; (**A**) Syncope, (**B**) Fatigue; (**C**) CKD, and (**D**) LVEF <40% in West Amhara comprehensive specialized hospitals, Amhara, Ethiopia, 2022.

In this study, we found that typical clinical symptom complaints during primary admission highly predicted the recurrence rate of ACS. The recurrence-free survival rate at the end of the follow-up period was higher among patients who had not complained of syncope during their initial admission compared with those who had complained of it (83.2% and 63.6%, respectively) (Figure 4A).

Another significant predictor was baseline fatigue symptoms. At the end of the follow-up, 83.3% of patients without complaints of fatigue survived, while only 65.2% of patients who experienced fatigue survived (Figure 4B).

Patients who had a history or comorbidity of chronic kidney disease also had a significant difference, as shown in the figure below. ACS patients with a history of CKD had a considerably lower survival rate than those without CKD history, with rates of 40% and 84.7%, respectively (Figure 4C). Patients with ACS who exhibited a reduced level of LVEF (<40%) also experienced a lower recurrence-free survival rate (69.8%) compared with those with an LVEF level of >40% (86.2%) (Figure 4D).

**Figure 5:**
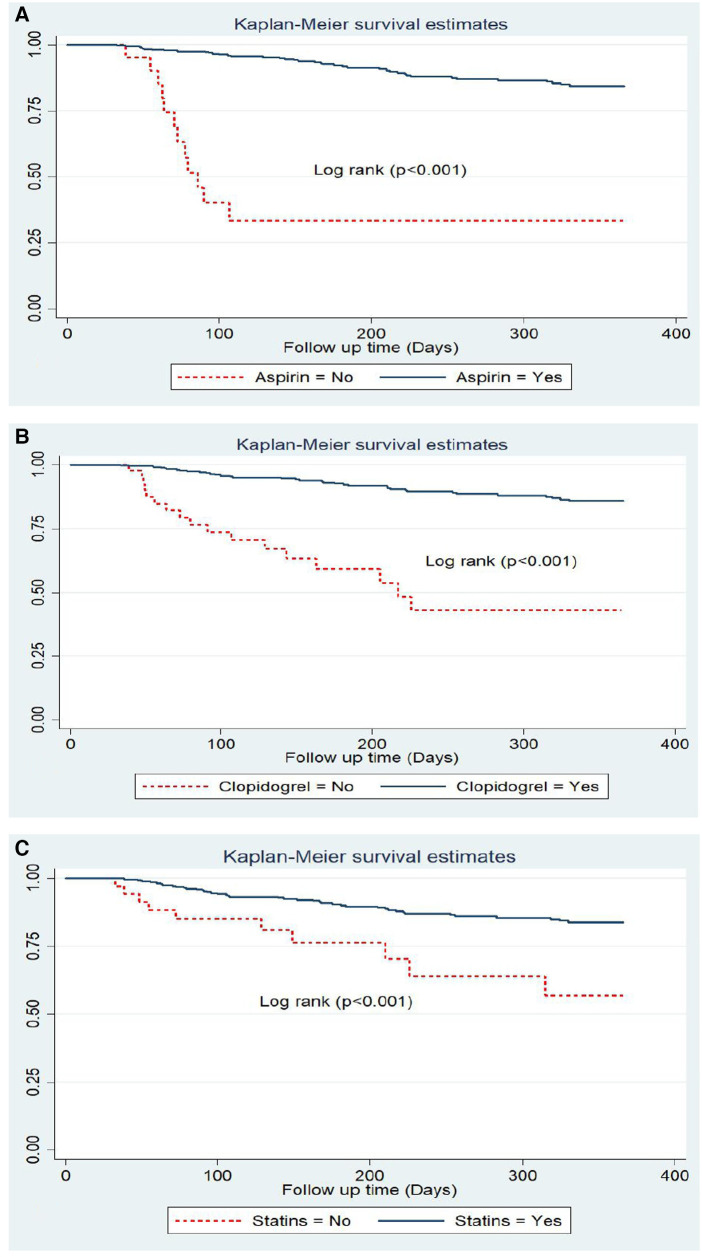
Kaplan Meier survival estimate by in hospital treatments; (**A**) aspirin, (**B**) clopidogrel and (**C**) statins among follow-up patients in West Amhara comprehensive specialized hospitals, amhara, Ethiopia, 2022.

Anti-ischemic treatments during primary admission also exhibited a significant difference in the recurrence-free survival rates. The survival rate of ACS patients without aspirin was significantly lower (33.8%) compared with those who received aspirin (84.5%). Another anti-ischemic drug is clopidogrel with a recurrence-free survival rate of 45% among prescribed patients and 85.8% among non-clopidogrel-prescribed ACS patients. Statins also had a significant difference as evidenced by a lower recurrence-free survival rate of 57.4% among the statins group compared with a rate of 84% among the non-statins group (Figure 5).

**Figure 6:**
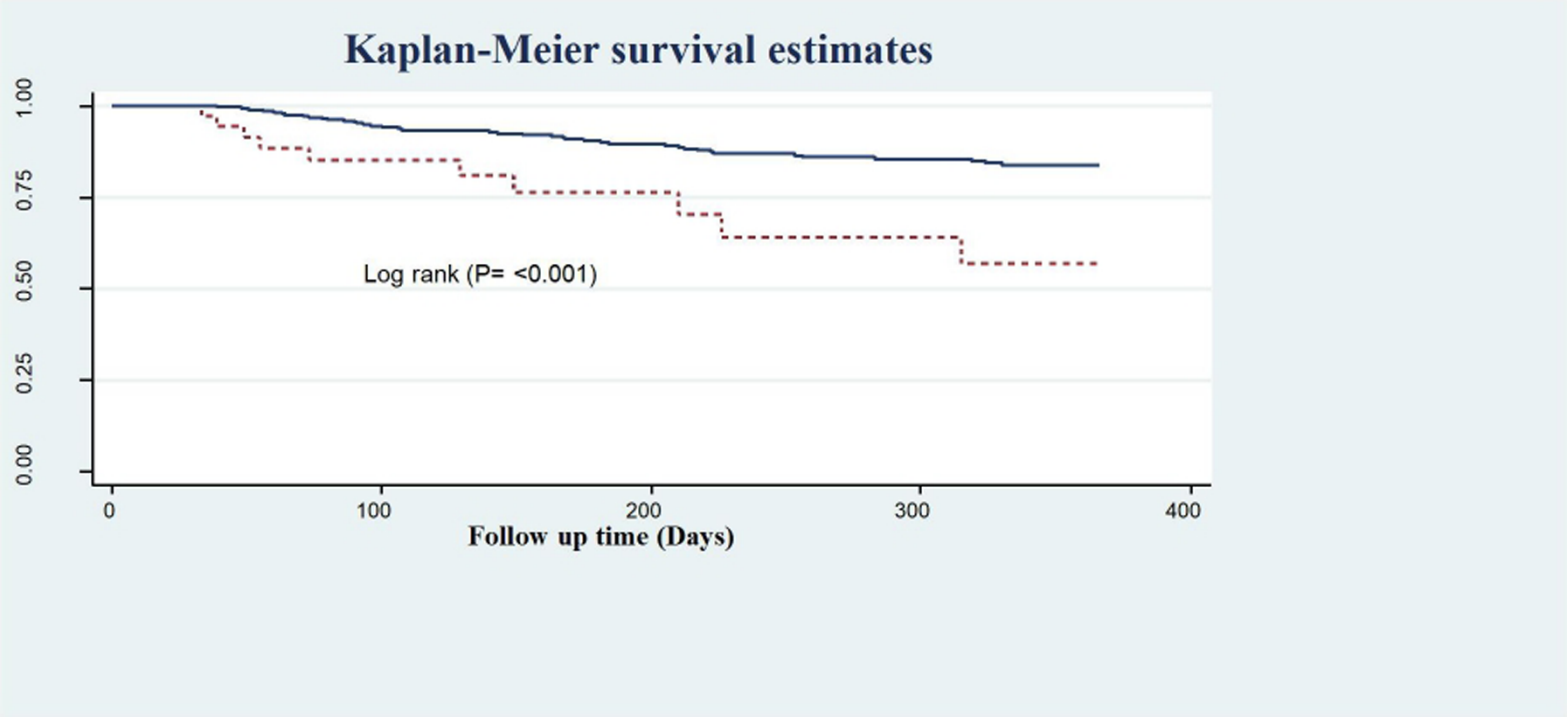
Kaplan–Meier survival estimate of LVEF of follow-up patients in West Amhara comprehensive specialized hospitals, Amhara, Ethiopia, 2022.

Another very important treatment provided upon discharge (medication taken home) was statins, which have been shown to effectively reduce lipid levels, as evidenced by the recurrence-free survival rates of 85.4% and 48.2% observed among those who were prescribed statins and those who were not, respectively (Figure 6).

### The goodness of fit of the final model

The goodness of fit of the final regression model was evaluated using the Schoenfeld residuals test and the estimate of Cox–Snell residuals drawn against the Nelson–Aalen cumulative hazard function. The global test of the proportional hazard assumption was conducted, revealing a lack of significance (the *p*-value for each variable ranges from 0.0811 to 0.9980, and the global test *p*-value of 0.5092). The Cox–Snell residual plot also indicated that the goodness of fit of the model was satisfied, as the hazard function follows the 45° line very closely. Therefore, we would conclude that the final model well aligns with the data. Unobserved heterogeneity in hospitals was also assessed using the gamma shared frailty model, and it was found that the theta value was approximately 0 (0.1344). Furthermore, the probability of the chi-square test was determined to be statistically insignificant (*p* = 0.236). Therefore, we concluded that the null hypothesis was not rejected, indicating that fixed effect models are suitable for analyzing the data.

### Model comparison

The log likelihood ratio, Akaike's information criterion (AIC), and Bayesian information criterion (BIC) were used to analyze the goodness of fit of the final model among semi-parametric and parametric models. Based on the available data, the Weibull PH regression model with the highest log likelihood, least AIC, and BIC was chosen as the final fitted analysis model (Table 6).

**Table 6:** Survival model selection with model fitness tests for a survival study on ACS patients in West Amhara comprehensive specialized hospitals, Amhara, Ethiopia, 2022.

Model fitness tests	Survival proportional hazard models			
	Cox	Exponential	Weibull	Gompertz
Log likely hood ratio	−222.83993	−127.04174	−116.91283	−123.2621
AIC	487.6799	298.0835	279.8257	292.5242
BIC	572.9704	387.4355	373.2392	385.9377

**Figure 7:**
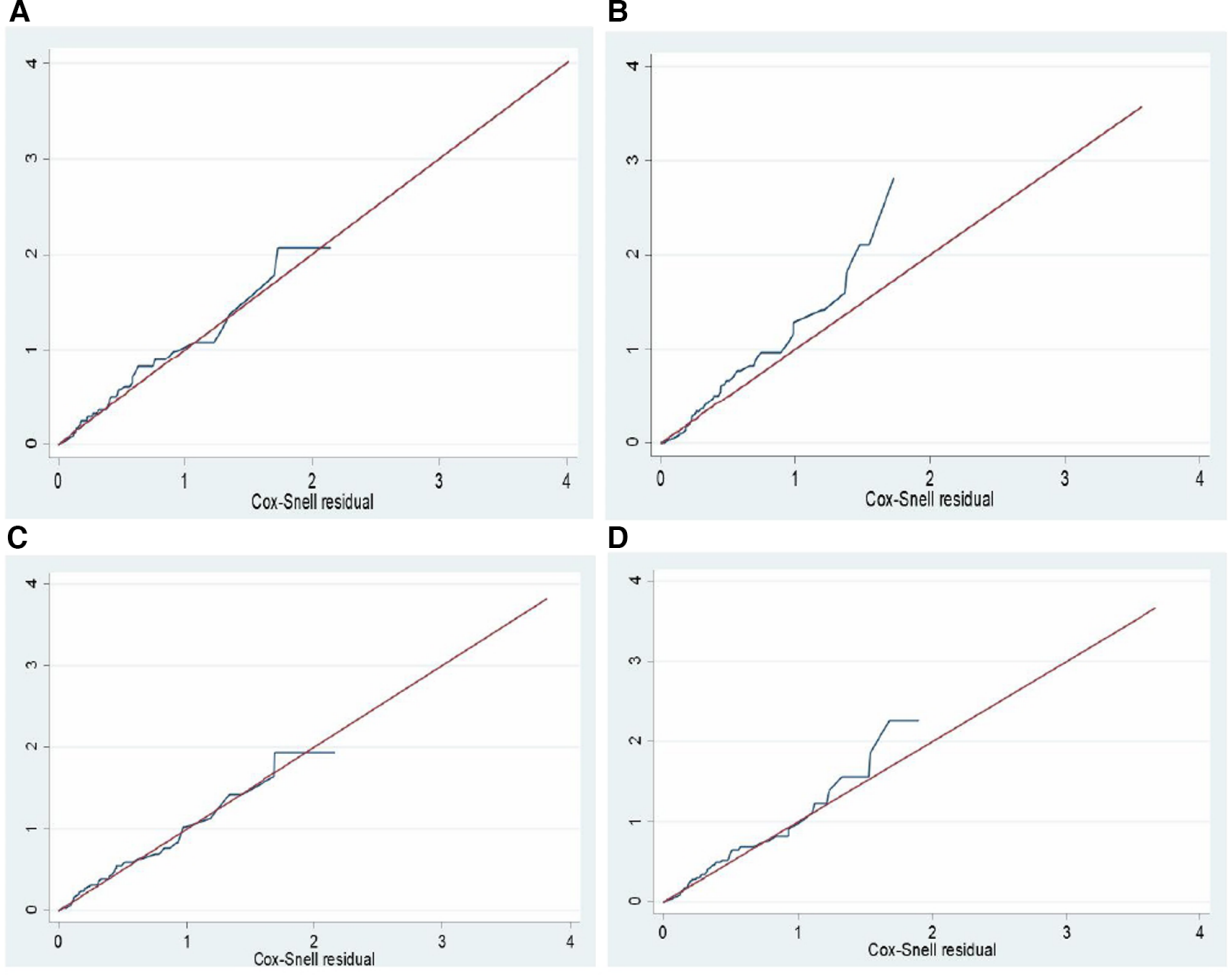
Nelson Aalen cumulative hazard function against Cox Snell residual multiple regression by; (**A**) cox regression, (**B**): exponential, (**C**): weibull, and (**D**): gompertz regression models for a survival study on ACS patients in West Amhara comprehensive specialized hospitals, Amhara, Ethiopia, 2022.

Figures 7A–D present a comparison between the Nelson–Aalen cumulative hazard function and Cox–Snell residual multivariable regressions, and the Weibull PH regression model was found to be the best-fit model.

### Predictors of recurrent acute coronary syndrome

Based on bivariable Weibull regression analysis, 19 variables were significant at *p*-value ≤ 0.25. Age, having symptoms of SOB, diaphoresis, syncope, and fatigue, history of comorbidities [stroke, DM, hypertension (HTN), CKD, HIV], altered level of LVEF, LV dysfunction, in-hospital treatments (aspirin, clopidogrel, statins, anticoagulant), and discharge medications (aspirin, clopidogrel, statins) were found to be significant predictors of recurrent ACS (Table 7). However, only eight variables were found to be predictors of recurrent ACS in a multivariable Weibull regression analysis, including syncope, fatigue, history of CKD, altered level of LVEF, in-hospital treatments (aspirin, statins, and anticoagulants), and discharge treatment (statins).

**Table 7:** Bivariable and multivariable Weibull regression analysis of predictors of recurrence among adult acute coronary syndrome patients in West Amhara comprehensive specialized hospitals, Amhara, Ethiopia, 2022.

Variables	Category	Outcome		CHR (95% CI)	*p*-value	AHR (95% CI)	*p*-value
		Censored, *n* (%)	Recurrent, *n* (%)				
Age (year)	18–44	53 (91.4)	5 (8.6)	1		1	
	45–64	191 (93.6)	13 (6.4)	0.63 (0.22–1.76)	0.376	0.33 (0.11–1.01)	0.053
	65–74	74 (86.0)	12 (14)	1.41 (0.50–4.01)	0.516	0.90 (0.28–2.84)	0.852
	≥75	58 (71.6)	23 (28.4)	2.92 (1.11–7.68)	0.030	1.42 (0.49–4.10)	0.518
Shortness of breathing	No	178 (91.3)	17 (8.7)	1		1	
	Yes	198 (84.6)	34 (15.4)	2.0 (1.12–3.57)	0.019	1.31 (0.67–2.60)	0.433
Diaphoresis	No	280 (86.7)	33 (10.5)	1		1	
	Yes	96 (82.1)	21 (17.9)	1.91 (1.09–3.33)	0.023	1.45 (0.72–2.920	0.301
Syncope	No	357 (88.6)	46 (11.4)	1		1	
	Yes	19 (73.1)	7 (16.9)	2.40 (1.08–5.32)	0.031	3.54 (1.31–9.55)	0.013*
Fatigue	No	346 (88.7)	44 (11.3)	1		1	
	Yes	30 (76.9)	9 (23.1)	2.83 (1.38–5.81)	0.005	5.23 (2.27–12.07)	<0.001**
History of stroke	No	347 (89.9)	39 (10.1)	1		1	
	Yes	29 (67.4)	14 (32.6)	3.96 (2.15–7.30)	0.000	1.12 (0.48–2.56)	0.815
History of DM	No	304 (89.4)	36 (10.6)	1		1	
	Yes	72 (80.9)	17 (19.1)	1.91 (1.07–3.40)	0.028	1.43 (0.72–2.81)	0.306
History of HTN	No	228 (89.8)	26 (10.1)	1		1	
	Yes	148 (84.6)	27 (15.4)	1.54 (0.897–2.63)	0.118	1.84 (0.93–3.65)	0.081
History of CKD	No	364 (89.7)	42 (10.3)	1		1	
	Yes	12 (52.2)	11 (47.8)	5.92 (3.04–11.51)	0.000	8.22 (3.03–22.27)	<0.001**
History of HIV	No	357 (89.5)	42 (10.5)	1		1	
	Yes	19 (63.3)	11 (36.7)	3.83 (1.97–7.44)	0.000	0.66 (0.24–1.80)	0.413
LVEF (%)	≥40	286 (90.8)	29 (9.2)	1		1	
	<40	90 (78.9)	24 (21.1)	2.58 (1.5–4.43)	0.001	2.34 (1.24–4.43)	0.009*
LV dysfunction	No	128 (94.8)	7 (5.2)	1		1	
	Yes	248 (84.4)	46 (15.6)	3.09 (1.4–6.84)	0.005	1.88 (0.69–5.11)	0.215
Aspirin	Yes	367 (90.0)	41 (10.0)	1		1	
	No	9 (42.9)	12 (57.1)	12.9 (6.68–24.99)	0.000	9.22 (3.56–23.90)	<0.001**
Clopidogrel	Yes	351 (90.7)	43 (9.3)	1		1	
	No	25 (76.2)	17 (23.8)	6.95 (3.88–12.46)	0.000	4.11 (1.75–9.63)	0.001*
Statins	Yes	350 (89.1)	43 (10.9)	1		1	
	No	26 (72.2)	10 (27.8)	3.29 (1.65–6.55)	0.001	2.74 (1.24–6.04)	0.012*
Anticoagulant	Yes	316 (89.5)	16 (11.1)	1		1	
	No	60 (78.9)	16 (21.1)	2.29 (1.27–4.12)	0.006	1.66 (0.85–3.24)	0.136
Aspirin at discharge	Yes	359 (89.8)	41 (10.3)	1		1	
	No	17 (58.6)	12 (41.4)	5.74 (3.0–10.94)	0.000	1.79 (0.72–4.43)	0.207
Clopidogrel at discharge	Yes	343 (90.7)	35 (9.3)	1		1	
	No	33 (64.7)	18 (35.3)	4.37 (2.48–7.72)	0.000	1.15 (0.48–2.73)	0.753
Statins at discharge	Yes	354 (90.3)	38 (9.7)	1		1	
	No	22 (59.5)	15 (40.5)	4.98 (2.74–9.06)	0.000	4.56 (2.14–9.72)	<0.001**

CHR, crude hazard ratio.

* Statistically significant at *p*-value < 0.05.

** Highly statistically significant at *p*-value < 0.001.

In this study, we found that typical clinical symptoms of ACS complaints during primary admission highly predict the recurrence rate of ACS. These symptoms were syncope and fatigue. Patients who had syncope complaints during primary admission were shown to have a 3.5-fold increased likelihood of experiencing recurrent ACS (AHR: 3.54, 95% CI: 1.31–9.55). ACS patients who presented with symptoms of fatigue also had more than five times higher hazard of developing recurrent ACS than those who had not (AHR: 5.23, 95% CI: 2.27–12.07).

Patients who had a history or comorbidity of chronic kidney disease had more than eight times the higher hazard of recurrent ACS compared with those who had not (AHR: 8.22, 95% CI: 3.03–22.27). ACS patients who had a decreased level of left ventricular ejection fraction (<40%) were also at a higher risk of developing recurrent ACS at a hazard of 2.34 times (AHR: 2.34, 95% CI: 1.24–4.43) when compared with those with LVEF of ≥40%.

Moreover, anti-ischemic treatments during primary admission were crucial to prevent secondary events such as recurrent ACS. ACS patients who did not take aspirin exhibited a higher hazard of developing recurrent ACS, which was 9.22 times compared with those who took aspirin (AHR: 9.22, 95% CI: 3.58–23.90).

Statins also had a significant difference as evidenced by a hazard ratio of 2.74 (AHR: 2.74, 95% CI: 1.24–6.04), which suggests a higher risk of recurrent ACS among those who were not prescribed with statins compared with those who had it. Another very important treatment at discharge (medication taken to home) was anticoagulants, which were found to have a strong association with a 4.56-fold increase (AHR: 4.56, 95% CI: 2.14–9.72) in the risk of developing recurrent ACS among those who did not take anticoagulants compared with those who did.

## Discussion

In this study, the rate of ACS recurrence 1 year after discharge was 12.35%. This finding is consistent with the previous reports conducted in developed nations such as the United States (9.6%) (30), Thailand (11.4%) (31), Australia (12.2%) (16), and Italy (10.1%) (32). One potential explanation could be due to the age similarity of the study population, which consisted of adults older than18 years. Another possible explanation could be the inclusion of all patients with ACS (STEMI and NSTEMI). In addition, the duration of follow-up in these studies was 1 year or longer.

However, the recurrent rate observed in this study is higher compared with a prior study conducted in Saudi Arabia (2.1%) (10), China (2.5%) (9), Japan (4.5%) (26), United States [(1.34%) (33), 4.7% (34), 8.47% (35)], Netherlands (8.0%) (12), and Brazil (9.14%) (13). The increased incidence of recurrent ACS may be attributed to the difference in the demographic characteristics of the study population. The study conducted in Saudi Arabia only included the recurrent ACS events that occurred during the in-hospital stay. Consequently, it may be associated with in-hospital intensive management that could reduce the recurrence rate. On the other hand, their duration of follow-up or time at risk was short, which may have decreased the incidence rate of recurrent ACS (10), whereas our study was conducted with a maximum follow-up period of 1 year, which may be the reason for the increased rate of ACS recurrence. Comparing our level of economic development level to that of these countries may also be a factor; in particular, it impedes our access to potent anti-ischemic and lipid-lowering drugs.

However, our results were lower than the 30% Re-ACS found in the French study (25). This previous study was conducted on an adult population aged 35–74 years, excluding individuals between the ages of 18 and 35 years. According to different literatures, this age group was at a lower risk of recurrent ACS. Therefore, neglecting this age group may result in a larger difference in the rate of recurrent ACS. Another reason could be that the French study included all events that occurred after discharge and during in-hospital treatments. Our study adopted a universal definition of recurrent myocardial infarction as recurrent ACS after 28 days of discharge, and incidents that occurred prior to this period were excluded. This could be the reason for the greater incidence rate in that study (25).

Second, we identified predictors of recurrent ACS, which is essential for a comprehensive understanding of patients with recurrent ACS and for taking secondary preventative measures. ACS recurrence was predicted by typical symptoms, such as altered levels of LVEF, CKD, and in-hospital and discharge medications, according to previous studies. Our multivariable Weibull regression analysis confirms these predictors.

Syncope and fatigue were found to be significant predictors of the recurrence rate of ACS in our study. These findings were supported by a French study in which typical symptoms of ACS at presentation were identified as predictors of Re-ACS (25).

As indicated by previous studies, the altered level of LVEF was an independent predictor of recurrent ACS. Our study also revealed that ACS patients with LVEF levels below 40% at their initial admission had a risk of recurrent ACS that was more than twice as high. This finding is consistent with a study conducted in Australia, which demonstrated that individuals with reduced LVEF had an increased risk of having recurrent ACS. On the contrary, studies conducted in China and Japan has indicated that a reduced level of LVEF was not significantly associated with recurrent ACS (9, 26).

Another predictor that was identified was the comorbidity of CKD, which was found to substantially predict the recurrence of ACS. A report from previous studies showed that patients who had a history of comorbidities were at higher risk of recurrent ACS (9, 10, 25, 31, 36). This finding is also consistent with previously published literature on the effect of CKD on patients with acute myocardial conditions. The reduction in GFR increases the risk of serious cardiovascular complications by approximately 20% (compared with subjects with normal renal function) (37). Our findings contribute to the existing knowledge by further supporting the association between chronic kidney disease and recurrent cardiovascular events.

This study found that evidence-based dual antiplatelet therapies (DAPT) were the major predictors of recurrent ACS. As reported in present and previous studies, in-hospital treatment with aspirin has demonstrated to reduce the risk of recurrent ACS. Individuals who did not take aspirin were at higher risk of developing Re-ACS. This was supported by previous studies in the United States and Saudi Arabia (10, 30). In contrast, a Chinese study showed that aspirin was not significantly associated with recurrent ACS (9). The pathophysiology of ACS was explained as the formation of a thrombus by platelet activation and aggregation. Guidelines recommended aspirin for such cases, and it works mainly by inhibiting the thromboxane A2 pathway and has additive anti-inflammatory effects. Non-enteric-coated, oral aspirin (162–325 mg) should be administered to all patients with ACS without contraindications as soon as possible after the presentation, and a maintenance dose of 81 mg (75–150 mg) aspirin per day should be continued indefinitely (38, 39).

The other guideline-recommended component of DAPT is clopidogrel, which is a P2Y12 receptor inhibitor broadly prescribed in our setup. Previous literature found that this medication was significantly associated with reducing the recurrence rate of ACS (9). Our study also agreed with the previous studies and found that patients who did not take clopidogrel were at higher risk of recurrent ACS. Individuals presenting with an ACS are recommended to be treated with a P2Y12 inhibitor in addition to aspirin, which is indicated as DAPT. Evidence shows that clopidogrel with or without aspirin is directed at limiting platelet adhesion and aggregation, which prevents additional thrombus formation. ACS patients are typically prescribed aspirin with clopidogrel (300 mg loading dose and 75 mg/day maintenance dose) for 3–12 months to improve their health (40).

Another predictor found in our study was statins. Statins are the main component of ACS treatment, which plays a crucial role in reducing cholesterol levels. Our study revealed that patients who were not prescribed statins during their hospital stay were at higher risk of Re-ACS compared with those who took statins. Other reports remarkably supported that statins were highly associated with reducing Re-ACS (9, 10, 30). However, a study conducted in Japan did not find a significant association between statins and the recurrent rate of ACS (26). In this study, we also found that statins prescription at discharge was highly associated with the recurrent rate of ACS.

The evidence showed that the long-term use of statins was beneficial for patients with ACS. Using these agents in combination with other anti-ischemic agents has a significant beneficial effect and should be administered concurrently to ACS patients. Statin therapy should be initiated early and continued long-term in all ACS patients irrespective of baseline LDL levels. In addition to lowering LDL levels, the potential benefits of statin therapy include plaque stabilization, improvement of endothelial function, reduced thrombogenicity, and reduced inflammation (41).

## Limitations and strengths of the study

The present study constitutes a secondary data review, wherein our focus was solely on only documented variables such as socio-demographics, typical symptoms of ACS, vital signs, diagnostic tests, comorbidities, and prescription of guideline-based medications. However, the exclusion of socio-demographic, behavioral, and nutritional status as key predictors of recurrence in earlier research was due to their insufficient data. These factors might be effectively examined through a prospective study. Furthermore, there is a need to evaluate whether the use of thrombolysis or revascularization, as well as coronary angiography procedures, have influenced the subsequent rates of recurrent infarction.

Despite the above potential limitations, the strength of this study is that it was conducted for an optimal period. This may increase the period of observation, which helps for better estimation of the post-discharge ACS recurrence rate. This study may also be used as a baseline for researchers in the context of resource-limited countries such as Ethiopia, since this is the first research specifically with this title as per the researcher’s level of search.

## Conclusion

This study found a higher rate of ACS recurrence compared with almost all studies reviewed. The main factors for this increased rate of Re-ACS were having a history of typical symptoms of ACS (syncope and fatigue), LVEF of <40%, history of comorbidity (CKD), and not taking in-hospital and discharge medications such as anti-ischemic and lipid-lowering treatments. Long-term intensive statin therapy may also be a reasonable therapeutic approach for those patients, and strengthening antiplatelet and lipid-lowering treatments are crucial.

## Data Availability

The raw data supporting the conclusions of this article will be made available by the authors, without undue reservation.

## References

[1] Lloyd-Jones D Adams RJ Brown TM Carnethon M Dai S De Simone G Heart disease and stroke statistics—2010 update: a report from the American Heart Association. Circulation. (2010) 121(7):e46–215. 20019324 10.1161/CIRCULATIONAHA.109.192667

[2] Gulati M Levy PD Mukherjee D Amsterdam E Bhatt DL Birtcher KK Writing Committee Membersetal/>. 2021 AHA/ACC/ASE/CHEST/SAEM/SCCT/SCMR guideline for the evaluation and diagnosis of chest pain: executive summary: a report of the American College of Cardiology/American Heart Association Joint Committee on Clinical Practice Guidelines. J Am Coll Cardiol. (2021) 78(22):2218–61. 10.1016/j.jacc.2021.07.052 34756652

[3] Di Vito L Niccoli G Porto I Vergallo R Gatto L Prati F Recurrent acute coronary syndrome and mechanisms of plaque instability. Int J Cardiol. (2017) 243:98–102. 10.1016/j.ijcard.2017.05.121 28601467

[4] Virani SS Alonso A Aparicio HJ Benjamin EJ Bittencourt MS Callaway CW Heart disease and stroke statistics—2021 update: a report from the American Heart Association. Circulation. (2021) 143(8):e254–743. 10.1161/CIR.0000000000000950 33501848 PMC13036842

[5] British Heart Foundation. *Global heart & circulatory diseases factsheet* (2022). Available at: https://www.bhf.org.uk/what-we-do/our-research/heart-statistics/heart-statistics-publications/cardiovascular-disease-statistics-2021 (Accessed March 5, 2022).

[6] World Health Organization. *The top 10 causes of death fact sheet* (2020). Available at: https://www.who.int/news-room/fact-sheets/detail/the-top-10-causes-of-death (Accessed March 20, 2022)

[7] World Health Organization. *World health statistics* (2021). Available at: https://www.who.int/data/stories/world-health-statistics-2021-a-visual-summary (Accessed March 10, 2022).

[8] Heidenreich PA Trogdon JG Khavjou OA Butler J Dracup K Ezekowitz MD Forecasting the future of cardiovascular disease in the United States: a policy statement from the American Heart Association. Circulation. (2011) 123(8):933–44. 10.1161/CIR.0b013e31820a55f5 21262990

[9] Song J Murugiah K Hu S Gao Y Li X Krumholz HM Incidence, predictors, and prognostic impact of recurrent acute myocardial infarction in China. Heart. (2020) 107(4):313–8. 10.1136/heartjnl-2020-317165 32938773 PMC7873426

[10] Al Saleh AS Alhabib KF Alsheik-Ali AA Sulaiman K Alfaleh H Alsaif S Predictors and impact of in-hospital recurrent myocardial infarction in patients with acute coronary syndrome: findings from Gulf RACE-2. Angiology. (2017) 68(6):508–12. 10.1177/0003319716674855 27784731

[11] Litovchik I Pereg D Shlomo N Vorobeichik D Beigel R Iakobishvili Z Characteristics and outcomes associated with 30-day readmissions following acute coronary syndrome 2000–2013: the Acute Coronary Syndrome Israeli Survey. Eur Heart J Acute Cardiovasc Care. (2019) 8(8):738–44. 10.1177/2048872618767997 29617148

[12] Kikkert WJ Zwinderman AH Vis MM Baan J Jr Koch KT Peters RJ Timing of mortality after severe bleeding and recurrent myocardial infarction in patients with ST-segment–elevation myocardial infarction. Circ Cardiovasc Interv. (2013) 6(4):391–8. 10.1161/CIRCINTERVENTIONS.113.000425 23941861

[13] Oliveira L Costa I Silva DGD Silva J Barreto-Filho JAS Almeida-Santos MA Readmission of patients with acute coronary syndrome and determinants. Arq Bras Cardiol. (2019) 113(1):42–9. 10.5935/abc.20190104 31271598 PMC6684196

[14] Belitardo JN Ayoub AC. Identification of readmission predictors in elderly patients with acute coronary syndrome. Int J Cardiovasc Sci. (2015) 28(2):139–47.

[15] Sangu PV Ranasinghe I Aliprandi Costa B Devlin G Elliot J Lefkovitz J Trends and predictors of rehospitalisation following an acute coronary syndrome: report from the Australian and New Zealand population of the Global Registry of Acute Coronary Events (GRACE). Heart. (2012) 98(23):1728–31. 10.1136/heartjnl-2012-302532 23010286

[16] Yudi MB Clark DJ Farouque O Andrianopoulos N Ajani AE Brennan A Trends and predictors of recurrent acute coronary syndrome hospitalizations and unplanned revascularization after index acute myocardial infarction treated with percutaneous coronary intervention. Am Heart J. (2019) 212:134–43. 10.1016/j.ahj.2019.02.013 31004916

[17] Mozaffarian D Benjamin EJ Go AS Arnett DK Blaha MJ Cushman M Executive summary: heart disease and stroke statistics—2015 update: a report from the American Heart Association. Circulation. (2015) 131(4):434–41. 10.1161/CIR.0000000000000157 25520374

[18] Southern DA Ngo J Martin BJ Galbraith PD Knudtson ML Ghali WA Characterizing types of readmission after acute coronary syndrome hospitalization: implications for quality reporting. J Am Heart Assoc. (2014) 3(5):e001046. 10.1161/JAHA.114.001046 25237046 PMC4323836

[19] Varwani MH Jeilan M Ngunga M Barasa A Outcomes in patients with acute coronary syndrome in a referral hospital in sub-Saharan Africa. Cardiovasc J Afr. (2019) 30(1):29–33. 10.5830/CVJA-2018-066 30534849

[20] Yadeta D Guteta S Alemayehu B Mekonnen D Gedlu E Benti H Spectrum of cardiovascular diseases in six main referral hospitals of Ethiopia. Heart Asia. (2017) 9(2):10–3. 10.1136/heartasia-2016-010829 PMC5827806 29492110

[21] Desta DM Nedi T Hailu A Atey TM Tsadik AG Asgedom SW Treatment outcome of acute coronary syndrome patients admitted to Ayder comprehensive specialized hospital, Mekelle, Ethiopia; a retrospective cross-sectional study. PLoS One. (2020) 15(2):1–17. 10.1371/journal.pone.0228953 PMC7018065 32053702

[22] Thygesen K Alpert JS Jaffe AS Chaitman BR Bax JJ Morrow DA Fourth universal definition of myocardial infarction (2018). Eur Heart J. (2018) 40(3):237–69. 10.1093/eurheartj/ehy462 30165617

[23] Webster AC Nagler EV Morton RL Masson P. Chronic kidney disease. Lancet. (2017) 389(10075):1238–52. 10.1016/S0140-6736(16)32064-5 27887750

[24] Mendis S Thygesen K Kuulasmaa K Giampaoli S Mähönen M Ngu Blackett K World Health Organization definition of myocardial infarction: 2008–09 revision. Int J Epidemiol. (2010) 40(1):139–46. 10.1093/ije/dyq165 20926369

[25] Machta S Gauthier V Ferrières J Montaye M Huo Yung Kai S Gbokou S Comparison of clinical profiles and care for patients with incident versus recurrent acute coronary syndromes in France: data from the MONICA registries. PLoS One. (2022) 17(2):e0263589. 10.1371/journal.pone.0263589 35157710 PMC8843220

[26] Nakatani D Sakata Y Suna S Usami M Matsumoto S Shimizu M Incidence, predictors, and subsequent mortality risk of recurrent myocardial infarction in patients following discharge for acute myocardial infarction. Circ J. (2012) 77(2):439–46. 10.1253/circj.CJ-11-1059 23075765

[27] Bogale K Mekonnen D Nedi T Woldu MA. Treatment outcomes of patients with acute coronary syndrome admitted to Tikur Anbessa Specialized Hospital, Addis Ababa, Ethiopia. Clin Med Insights Cardiol. (2019) 13(1):1179546819839417. 10.1177/1179546819839417 31024218 PMC6472164

[28] Zamani P Schwartz GG Olsson AG Rifai N Bao W Libby P Inflammatory biomarkers, death, and recurrent nonfatal coronary events after an acute coronary syndrome in the MIRACL study. Am Heart Assoc. (2013) 2(1):e003103. 10.1161/JAHA.112.003103 PMC3603244 23525424

[29] D'Ascenzo F De Filippo O Gallone G Mittone G Deriu MA Iannaccone M Machine learning-based prediction of adverse events following an acute coronary syndrome (PRAISE): a modelling study of pooled datasets. Lancet 2021 397(10270): 199–207. 33453782 10.1016/S0140-6736(20)32519-8

[30] Thune JJ Signorovitch JE Kober L McMurray JJ Swedberg K Rouleau J Predictors and prognostic impact of recurrent myocardial infarction in patients with left ventricular dysfunction, heart failure, or both following a first myocardial infarction. Eur J Heart Fail. (2011) 13(2):148–53. 10.1093/eurjhf/hfq194 21037250

[31] Chinwong D Patumanond J Chinwong S Siriwattana K Gunaparn S Hall JJ Clinical indicators for recurrent cardiovascular events in acute coronary syndrome patients treated with statins under routine practice in Thailand: an observational study. BMC Cardiovasc Disord. (2015) 15(1):1–9. 10.1186/s12872-015-0052-y 26076586 PMC4467053

[32] Galasso G De Angelis E Silverio A Di Maio M Cancro FP Esposito L Predictors of recurrent ischemic events in patients with ST-segment elevation myocardial infarction. Am J Cardiol. (2021) 159:44–51. 10.1016/j.amjcard.2021.08.019 34503819

[33] Lemor A Hernandez GA Patel N Blumer V Sud K Cohen MG Predictors and etiologies of 30-day readmissions in patients with non-ST-elevation acute coronary syndrome. Catheter Cardiovasc Interv. (2019) 93(3):373–9. 10.1002/ccd.27838 30280472

[34] Gilpin E Ricou F Dittrich H Nicod P Henning H Ross J Jr. Factors associated with recurrent myocardial infarction within one year after acute myocardial infarction. Am Heart J. (1991) 121(2):457–65. 10.1016/0002-8703(91)90712-Q 1990749

[35] Khot UN Johnson MJ Wiggins NB Lowry AM Rajeswaran J Kapadia S Long-term time-varying risk of readmission after acute myocardial infarction. J Am Heart Assoc. (2018) 7(21):e009650. 10.1161/JAHA.118.0096650 30375246 PMC6404216

[36] Nair R Johnson M Kravitz K Huded C Rajeswaran J Anabila M Characteristics and outcomes of early recurrent myocardial infarction after acute myocardial infarction. J Am Heart Assoc. (2021) 10(16):e019270. 10.1161/JAHA.120.019270 34333986 PMC8475017

[37] Franczyk-Skóra B Gluba A Banach M Rysz J. State of the art paper treatment of non-ST-elevation myocardial infarction and ST-elevation myocardial infarction in patients with chronic kidney disease. Arch Med Sci. (2013) 9(6):1019–27. 10.5114/aoms.2013.39792 24482645 PMC3902722

[38] Roffi M Patrono C Collet JP Mueller C Valgimigli M Andreotti F Linee guida ESC 2015 per il trattamento delle sindromi coronariche acute nei pazienti senza sopraslivellamento persistente del tratto ST alla presentazione: task force per il trattamento delle sindromi coronariche acute nei pazienti senza sopraslivellamento persistente del tratto ST alla presentazione della, società europea di cardiologia (ESC). G Ital Cardiol. (2016) 17(10):831–72. 10.1093/eurheartj/ehv320 27869901

[39] Bittl JA Baber U Bradley SM Wijeysundera DN. 2016 ACC/AHA guideline focused update on duration of dual antiplatelet therapy in patients with coronary artery disease: a report of the American College of Cardiology/American Heart Association Task Force on Clinical Practice Guidelines. Circulation. (2016) 134(10):e123–55. 10.1161/CIR.0000000000000404 27026019

[40] Yusuf S Zhao F Mehta SR Chrolavicius S Tognoni G Fox KK. Effects of clopidogrel in addition to aspirin in patients with acute coronary syndromes without ST-segment elevation. N Engl J Med. (2001) 345(7):494–502. 10.1056/NEJMoa010746 11519503

[41] Mukherjee D Fang J Chetcuti S Moscucci M Kline-Rogers E Eagle KA. Impact of combination evidence-based medical therapy on mortality in patients with acute coronary syndromes. Circulation. (2004) 109(6):745–9. 10.1161/01.CIR.0000112577.69066.CB 14970110

